# Active Semi-Supervised Learning Method with Hybrid Deep Belief Networks

**DOI:** 10.1371/journal.pone.0107122

**Published:** 2014-09-10

**Authors:** Shusen Zhou, Qingcai Chen, Xiaolong Wang

**Affiliations:** 1 School of Information and Electrical Engineering, Ludong University, Yantai, Shandong, China; 2 Shenzhen Graduate School, Harbin Institute of Technology, Shenzhen, Guangdong, China; Politehnica University of Bucharest, Romania

## Abstract

In this paper, we develop a novel semi-supervised learning algorithm called active hybrid deep belief networks (AHD), to address the semi-supervised sentiment classification problem with deep learning. First, we construct the previous several hidden layers using restricted Boltzmann machines (RBM), which can reduce the dimension and abstract the information of the reviews quickly. Second, we construct the following hidden layers using convolutional restricted Boltzmann machines (CRBM), which can abstract the information of reviews effectively. Third, the constructed deep architecture is fine-tuned by gradient-descent based supervised learning with an exponential loss function. Finally, active learning method is combined based on the proposed deep architecture. We did several experiments on five sentiment classification datasets, and show that AHD is competitive with previous semi-supervised learning algorithm. Experiments are also conducted to verify the effectiveness of our proposed method with different number of labeled reviews and unlabeled reviews respectively.

## Introduction

Recently, more and more people write reviews and share opinions on the World Wide Web, which present a wealth of information on products and services [1]. These reviews will not only help other users make better judgements but they are also useful resources for manufacturers of products to keep track and manage customer opinions [2]. However, there are large amounts of reviews for every topic, it is difficult for a user to manually learn the opinions of an interesting topic. Sentiment classification, which aims to classify a text according to the expressed sentimental polarities of opinions such as ‘*positive*’ or ‘*negative*’, ‘*thumb up*’ or ‘*thumb down*’, ‘*favorable*’ or ‘*unfavorable*’ [3], can facilitate the investigation of corresponding products or services.

In order to learn a good text classifier, a large number of labeled reviews are often needed for training [4]. However, labeling reviews is often difficult, expensive or time consuming [5]. On the other hand, it is much easier to obtain a large number of unlabeled reviews, such as the growing availability and popularity of online review sites and personal blogs [6]. In recent years, a new approach called semi-supervised learning, which uses large amount of unlabeled data together with labeled data to build better learners [7], has been developed in the machine learning community.

There are several works have been done in semi-supervised learning for sentiment classification, and have get competitive performance [3], [8]–[10]. However, most of the existing semi-supervised learning methods are still far from satisfactory. As shown by several researchers [11], [12], deep architecture, which composed of multiple levels of non-linear operations, is expected to perform well in semi-supervised learning because of its capability of modeling hard artificial intelligent tasks. Deep belief networks (DBN) is a representative deep learning algorithm achieving notable success for text classification, which is a directed belief nets with many hidden layers constructed by restricted Boltzmann machines (RBM), and refined by a gradient-descent based supervised learning [12]. Ranzato and Szummer [13] propose an algorithm to learn text document representations based on semi-supervised auto-encoders that are combined to form a deep network. Zhou et al. [10] propose a novel semi-supervised learning algorithm to address the semi-supervised sentiment classification problem with active learning. Socher et al. [14] introduce a novel machine learning framework based on recursive autoencoders for sentence-level prediction of sentiment label distributions. Socher et al. [15] introduce the recursive neural tensor network for semantic compositionality over a sentiment treebank. The key issue of traditional DBN is the efficiency of RBM training. Convolutional neural networks (CNN), which are specifically designed to deal with the variability of two dimensional shapes, have had great success in machine learning tasks and represent one of the early successes of deep learning [16]. Desjardins and Bengio [17] adapt RBM to operate in a convolutional manner, and show that the convolutional RBM (CRBM) are more efficient than standard RBM.

CRBM has been applied successfully to a wide range of visual and audio recognition tasks [18], [19]. Though the success of CRBM in addressing two dimensional issues, there is still no published research on the using of CRBM in textual information processing. In this paper, we propose a novel semi-supervised learning algorithm called active hybrid deep belief networks (AHD), to address the semi-supervised sentiment classification problem with deep learning. AHD is an active learning method based on deep architecture, which the bottom layers are constructed by RBM, and the upper layers are constructed by CRBM, then the whole constructed deep architecture is fine tuned by a gradient-descent based supervised learning based on an exponential loss function.

## Hybrid Deep Belief Networks Method

### Problem formulation

The sentiment classification dataset composed of many review documents, each review document composed of a bag of words. To classify these review documents using corpus-based approaches, we need to preprocess them in advance. The preprocess method for these reviews is similar with [9], [10]. We tokenize and downcase each review and represent it as a vector of unigrams, using binary weight equal to 1 for terms present in a vector. Moreover, the punctuations, numbers, and words of length one are removed from the vector. Finally, we combine all the words in the dataset, sort the vocabulary by document frequency and remove the top 1.5%, because many of these high document frequency words are stopwords or domain specific general-purpose words.

After preprocess, each review can be represented as a vector of binary weight 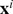. If the 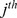 word of the vocabulary is in the 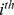 review, 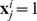; otherwise, 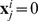. Then the dataset can be represented as a matrix: 
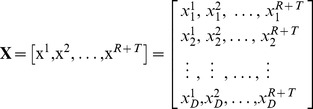
(1)where 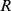 is the number of training reviews, 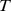 is the number of test reviews, 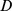 is the number of feature words in the dataset. Every column of 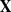 corresponds to a sample 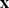, which is a representation of a review. A sample that has all features is viewed as a vector in 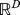, where the 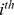 coordinate corresponds to the 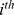 feature.

The 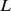 labeled reviews are chosen randomly from 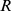 training reviews, or chosen actively by active learning, which can be seen as: 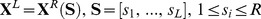
(2)where 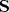 is the index of selected training reviews to be labeled manually.

The 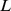 labels correspond to 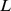 labeled training reviews is denoted as: 
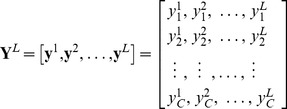
(3)where 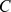 is the number of classes. Every column of 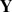 is a vector in 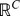, where the 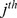 coordinate corresponds to the 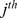 class. 
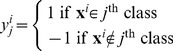
(4)


For example, if a review 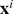 is positive, 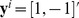; otherwise, 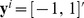.

We intend to seek the mapping function 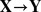 using the 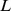 labeled data and all unlabeled data. After training, we can determine 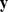 using the mapping function when a new sample 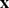 comes.

### Architecture of HDBN

**Figure 1:**
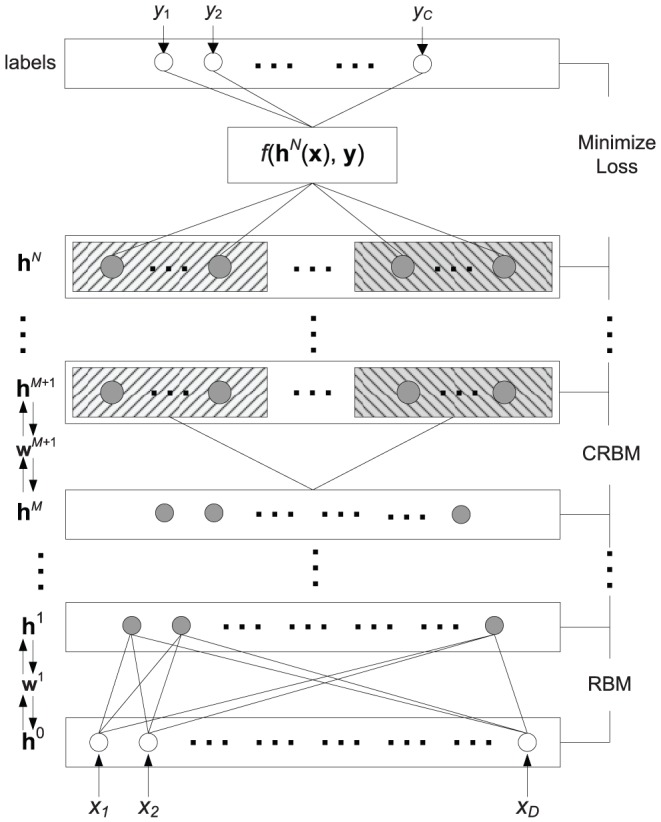
Architecture of HDBN.

In this part, we propose a novel semi-supervised learning method HDBN to address the sentiment classification problem. The sentiment datasets have high dimension (about 10,000), and computation complexity of convolutional calculation is relatively high, so we use RBM to reduce the dimension of review with normal calculation firstly. Fig. 1 shows the deep architecture of HDBN, a fully interconnected directed belief nets with one input layer 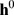, 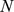 hidden layers 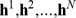, and one label layer at the top. The input layer 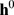 has 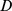 units, equal to the number of features of sample review 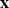. The hidden layer has 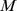 layers constructed by RBM and 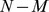 layers constructed by CRBM. The label layer has 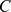 units, equal to the number of classes of label vector 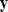. The numbers of hidden layers and the number of units for hidden layers, currently, are pre-defined according to the experience or intuition. The seeking of the mapping function 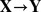, here, is transformed to the problem of finding the parameter space 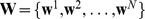 for the deep architecture.

The training of the HDBN can be divided into two stages:

1. HDBN is constructed by greedy layer-wise unsupervised learning using RBMs and CRBMs as building blocks. 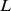 labeled data and all unlabeled data are utilized to find the parameter space 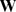 with 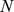 layers.

2. HDBN is trained according to the exponential loss function using gradient descent based supervised learning. The parameter space 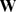 is refined using 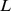 labeled data.

### Unsupervised learning

As show in Fig. 1 , we construct HDBN layer by layer using RBMs and CRBMs, the details of RBM can be seen in [12]. CRBM is introduced below.

**Figure 2:**
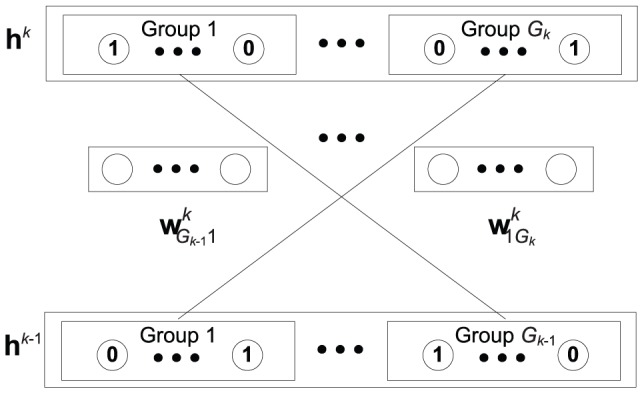
Architecture of CRBM.

The architecture of CRBM can be seen in Fig. 2, which is similar to RBM, a two-layer recurrent neural network in which stochastic binary input groups are connected to stochastic binary output groups using symmetrically weighted connections. The top layer represents a vector of stochastic binary hidden feature 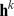 and the bottom layer represents a vector of binary visible data 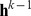, 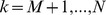. The 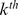 layer consists of 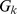 groups, where each group consists of 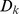 units, resulting in 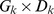 hidden units. The layer 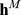 is consist of 1 group and 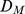 units. 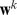 is the symmetric interaction term connecting corresponding groups between data 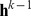 and feature 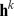. However, comparing with RBM, the weights of CRBM between the hidden and visible groups are shared among all locations [18], and the calculation is operated in a convolutional manner [17].

We define the energy of the state 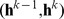 as: 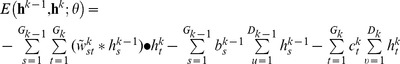
(5)where 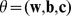 are the model parameters: 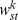 is a filter between unit 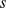 in the layer 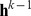 and unit 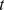 in the layer 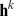, 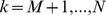. The dimension of the filter 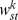 is equal to 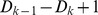. 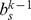 is the 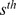 bias of layer 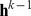 and 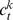 is the 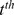 bias of layer 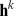. A tilde above an array (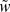) denote flipping the array, 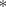 denote valid convolution, and 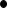 denote element-wise product followed by summation, i.e., 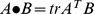 [18].

Gibbs sampler can be performed based on the following conditional distribution.

The probability of turning on unit 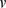 in group 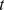 is a logistic function of the states of 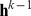 and 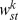: 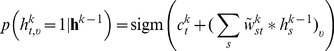
(6)


The probability of turning on unit 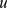 in group 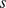 is a logistic function of the states of 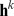 and 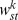: 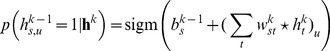
(7)where the logistic function is: 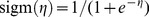
(8)


A star 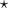 denotes full convolution.

The convolution computation can extract the information of text effectively based on deep architecture, although it needs more computation time.

### Supervised learning

In HDBN, we construct the deep architecture using all labeled reviews with unlabeled reviews by inputting them one by one from layer 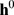. The deep architecture is constructed layer by layer from bottom to top, and each time, the parameter space 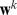 is trained by the calculated data in the 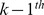 layer.

According to the 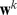 calculated by RBM and CRBM, the layer 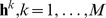 can be computed as following when a sample 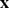 inputs from layer 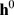: 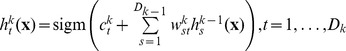
(9)


When 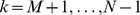, the layer 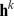 can be represented as: 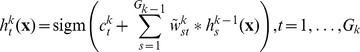
(10)


The parameter space 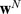 is initialized randomly, just as backpropagation algorithm. 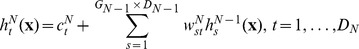
(11)


After greedy layer-wise unsupervised learning, 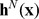 is the representation of 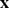. Then we use 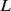 labeled reviews to refine the parameter space **W** for better discriminative ability. This task can be formulated as an optimization problem: 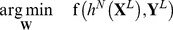
(12)where 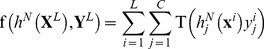
(13)and the loss function is defined as 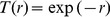
(14)


We use gradient-descent through the whole HDBN to refine the weight space. In the supervised learning stage, the stochastic activities are replaced by deterministic, real valued probabilities.

### Classification using HDBN

The training procedure of HDBN is given in Table 1. For the training of HDBN architecture, the parameters are random initialized with normal distribution. All the reviews in the dataset are used to train the HDBN with unsupervised learning. After training, we can determine the label of the new data through: 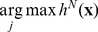
(15)


**Table 1:** Algorithm of HDBN.

**Input:**
data 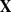 , 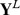
number of training data 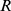 ; number of test data 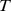 ;
number of layers 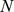 ; number of epochs 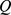 ;
number of units in every hidden layer 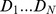 ;
number of groups in every convolutional hidden layer 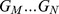 ;
hidden layer 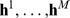 ;
convolutional hidden layer 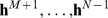 ;
parameter space 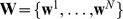 ;
biases 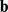 , 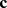 ; momentum 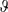 and learning rate 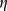 ;
**Output:**
deep architecture with parameter space 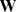
1. Greedy layer-wise unsupervised learning
**for** 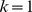 ; 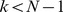 ; 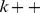 **do**
**for** 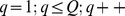 **do**
**for** 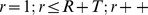 **do**
Calculate the non-linear positive and negative phase:
**if** 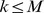 **then**
Normal calculation.
**else**
Convolutional calculation according to Eq. 6 and Eq. 7.
**end if**
Update the weights and biases:
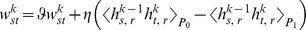
**end for**
**end for**
**end for**
2. Supervised learning based on gradient descent
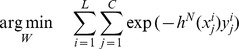

## Active Hybrid Deep Belief Networks Method

### AHD description

Given an unlabeled pool 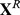 and an initial labeled data set 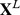 (one positive, one negative), the AHD architecture 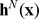 will decide which instance in 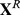 to query next. Then the parameters of 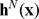 are adjusted after new reviews are labeled and inserted into the labeled data set 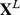. We choose the reviews that are near the separating hyperplane as the labeled training data.

When HDBN is trained by 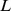 labeled data and all unlabeled data, the parameters of deep architecture are adjusted, 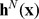 is the representation of 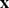. Given an unlabeled pool 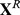, the next unlabeled instance to be queried are chosen according to the location of 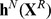. For review document, there are only 2 classes (*positive* or *negative*), so the dimension of 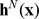 is 2, the classes separation line is 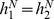. The distance between a point 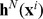 and separation line is: 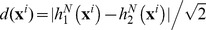
(16)


The selected training reviews to be labeled manually are given by: 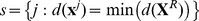
(17)


### Classification using AHD

The training procedure of AHD is given in Table 2. The training set 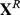 can be seen as an unlabeled pool. We randomly select one positive and one negative reviews in the pool to input as the initial labeled dataset 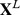 that are used for supervised learning. The iteration times 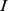 and the number of active choosing data 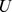 for each iteration can be set manually based on the number of labeled reviews in the experiment.

**Table 2:** Algorithm of AHD.

**Input:**
data 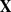 , 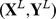 (one positive and one negative)
number of training data 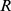
number of iterations 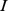
number of active choosing data for every iteration 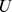
parameter space 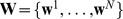
**Output:**
deep architecture with parameter space 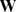
**for** 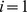 ; 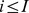 ; 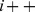 **do**
Train HDBN with labeled dataset 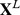 and all unlabeled data in 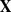 .
Choose 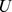 reviews which near the separating line from train dataset 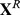 through Eq. 17.
Add 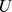 reviews into the labeled data set 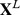 .
**end for**
Train HDBN with labeled dataset 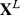 and all unlabeled data in 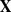 .

For each iteration, the HDBN architecture is trained by all the unlabeled reviews and labeled reviews in existence with unsupervised learning and supervised learning firstly. Then 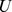 reviews are chosen from the unlabeled pool based on the distance of these review mapping results from the separating line. At last, these 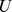 reviews are labeled manually and added to the labeled dataset 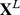. For the next iteration, the HDBN architecture can be re-trained by all reviews with unsupervised learning and all labeled reviews with the new increased labeled dataset 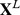. At last, HDBN architecture is retrained by all the reviews with unsupervised learning and existing labeled reviews with supervised learning.

After active training, we can use the Eq. 15 to determine the label of the new data. The purpose of active learning is choose more useful label data to train the deep architecture, which can use fewer label data to train better classifier.

## Experiments

### Experimental setup

We evaluate the performance of the proposed HDBN and AHD method using five sentiment classification datasets. The first dataset is MOV [20], which is a classical movie review dataset. The other four datasets contain products reviews come from the multi-domain sentiment classification corpus, including books (BOO), DVDs (DVD), electronics (ELE), and kitchen appliances (KIT) [21]. Each dataset contains 1,000 positive and 1,000 negative reviews.

The experimental setup is same as [9] and [10]. We divide the 2,000 reviews into ten equal-sized folds randomly, maintaining balanced class distributions in each fold. Half of the reviews in each fold are random selected as training data and the remaining reviews are used for test. Only the reviews in the training data set are used for the selection of labeled reviews by active learning. All the algorithms are tested with cross-validation.

We compare the classification performance of HDBN with four representative semi-supervised learning methods, i.e., semi-supervised spectral learning (Spectral) [22], transductive SVM (TSVM) [23], deep belief networks (DBN) [12], and personal/impersonal views (PIV) [3]. Spectral learning, TSVM methods are two baseline methods for sentiment classification. DBN [12] is the classical deep learning method proposed recently. PIV [3] is a new sentiment classification method proposed recently.

We also compare the classification performance of AHD with three representative active semi-supervised learning methods, i.e., active learning (Active) [24], mine the easy classify the hard (MECH) [9], and active deep networks (ADN) [10]. Active learning [24] is a baseline active learning method for sentiment classification. MECH [9] and ADN [10] are two new active learning method for sentiment classification proposed recently.

### Performance of HDBN

The HDBN architecture used in all our experiments have 2 normal hidden layer and 1 convolutional hidden layer, every hidden layer has different number of units for different sentiment datasets. The deep structure used in our experiments for different datasets can be seen in Table 3. For example, the HDBN structure used in MOV dataset experiment is 100-100-4-2, which represents the number of units in 2 normal hidden layers are 100, 100 respectively, and in output layer is 2, the number of groups in 1 convolutional hidden layer is 4. The number of unit in input layer is the same as the dimensions of each datasets. For greedy layer-wise unsupervised learning, we train the weights of each layer independently with the fixed number of epochs equal to 30 and the learning rate is set to 0.1. The initial momentum is 0.5 and after 5 epochs, the momentum is set to 0.9. For supervised learning, we run 30 epochs, three times of linear searches are performed in each epoch.

**Table 3:** HDBN structure used in experiment.

Dataset	Structure
MOV	100-100-4-2
KIT	50-50-3-2
ELE	50-50-3-2
BOO	50-50-5-2
DVD	50-50-5-2

The test accuracies in cross validation for five datasets and five methods with semi-supervised learning are shown in Table 4. The results of previous two methods are reported by [9]. The results of DBN method are reported by [10]. Li et al. [3] reported the results of PIV method. The result of PIV on MOV dataset is empty, because [3] did not report it. HDBN is the proposed method.

**Table 4:** Test accuracy with 100 labeled reviews for semi-supervised learning.

Type	MOV	KIT	ELE	BOO	DVD
Spectral	67.3	63.7	57.7	55.8	56.2
TSVM	68.7	65.5	62.9	58.7	57.3
DBN	71.3	72.6	73.6	64.3	66.7
PIV	–	**78.6**	70.0	60.1	49.5
HDBN	**72.2**	74.8	**73.8**	**66.0**	**70.3**

Through Table 4, we can see that HDBN gets most of the best results except on KIT dataset, which is just slight worse than PIV method. However, the preprocess of PIV method is much more complicated than HDBN, and the PIV results on other datasets are much worse than HDBN method. HDBN method is adjusted by DBN, all the experiment results on five datasets for HDBN are better than DBN. This could be contributed by the convolutional computation in HDBN structure, and proves the effectiveness of our proposed method.

### Performance of AHD

To evaluate the performance of AHD, we compare its results with several previous active learning methods for sentiment classification. The architectures used in this experiments can be seen in Table 3. We perform active learning for 5 iterations. In each iteration, we select and label 20 of the most uncertain reviews, and then retrain the deep architecture on all of the unlabeled reviews and labeled reviews annotated so far. After 5 iterations, 100 labeled reviews are used for training.

The test accuracies in cross validation for five datasets and four methods with active semi-supervised learning are shown in Table 5. The results of previous two methods are reported by [9]. The results of ADN method are reported by [10]. AHD is the proposed active learning method in this paper. Through Table 5, we can see that the results of AHD is better than Active and MECH methods, and competitive with ADN method. Because ADN and AHD methods are both deep learning method, these results prove that deep architecture is good for sentiment classification.

**Table 5:** Test accuracy with 100 labeled reviews for active semi-supervised learning.

Type	MOV	KIT	ELE	BOO	DVD
Active	68.9	68.1	63.3	58.6	58.0
MECH	76.2	74.1	70.6	62.1	62.7
ADN	**76.3**	**77.5**	**76.8**	69.0	71.6
AFD	75	77	**76.8**	**70.1**	**73.7**

### Performance with variance of unlabeled data

To verify the contribution of unlabeled reviews for our proposed method, we did several experiments with fewer unlabeled reviews and 100 labeled reviews. We use HDBN method in this part, considering AHD method choose the reviews need to label from an unlabeled pool, it is unfair to compare the performance of AHD when the size of unlabeled pool is different.

**Figure 3:**
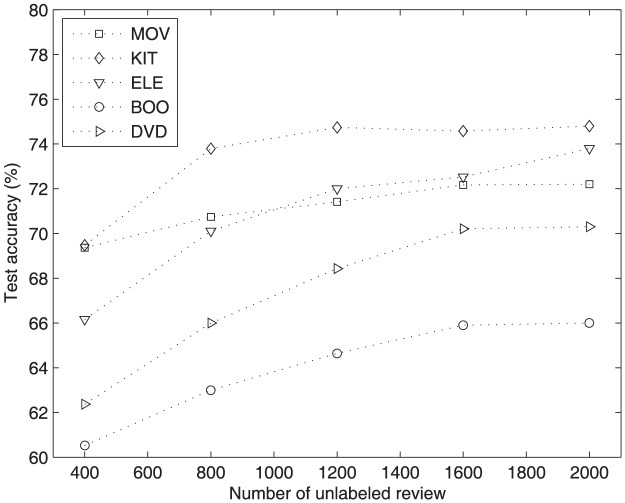
Test accuracy of HDBN with different number of unlabeled reviews on five datasets.

The test accuracies of HDBN with different number of unlabeled reviews and 100 labeled reviews on five datasets are shown in Fig. 3. The architectures for HDBN used in this experiment can be seen in Table 3. We can see that the performance of HDBN is much worse when just using 400 unlabeled reviews. However, when using more than 1200 unlabeled reviews, the performance of HDBN is improved obviously. For most of review datasets, the accuracy of HDBN with 1200 unlabeled reviews is close to the accuracy with 1600 and 2000 unlabeled reviews. This proves that HDBN can get competitive performance with just few labeled reviews and appropriate number of unlabeled reviews. Considering the much time needed for training with more unlabeled reviews and less accuracy improved for HDBN method, we suggest using appropriate number of unlabeled reviews in real application.

### Performance with variance of labeled data

To verify the contribution of labeled reviews for our proposed method, we did several experiments with different number of labeled reviews on five datasets. To compare the active learning performance with ADN [10], we use AHD method in this experiment, all the experimental setting are same as ADN. The architectures for AHD used in this experiment can be seen in Table 3.

**Figure 4:**
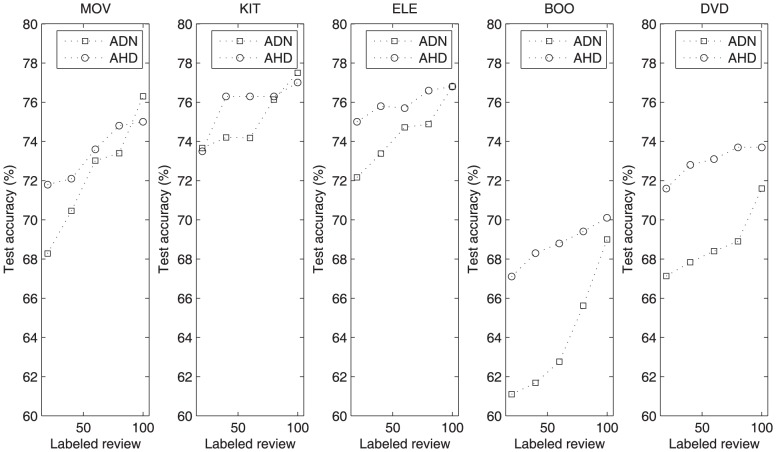
Test accuracy of ADN and AHD with different number of labeled reviews on five datasets.

The test accuracies of ADN and AHD with different number of labeled reviews on five datasets are shown in Fig. 4. We can see that the performance of AHD is better than ADN for most of the experimental setting, although they are both based on the DBN method. This proves that the convolutional computation has better performance than the normal computation in the deep architecture for sentiment classification. We can also see that both ADN and AHD can get high accuracy even with just 20 labeled reviews for training. This proves the effect of deep learning method for semi-supervised learning with very few labeled reviews.

## Conclusions

In this paper, we propose a novel semi-supervised learning method, AHD, to address the sentiment classification problem with a small number of labeled reviews. AHD seamlessly incorporate convolutional computation into the DBN architecture, and use CRBM to abstract the review information effectively. One promising property of AHD is that it can effectively use the distribution of large amount of unlabeled data, together with few label information in a unified framework. In particular, AHD can greatly reduce the dimension of reviews through RBM and abstract the information of reviews through the cooperate of RBM and CRBM. Then an exponential loss function is used to refine the constructed deep architecture with few label information. Moreover, it can choose the review to be labeled actively, improve the performance of deep architecture effectively.

Experiments conducted on five sentiment datasets demonstrate that AHD outperforms most of previous methods and is competitive with DBN based method, which demonstrates the performance of deep architecture for sentiment classification. Experiments are also conducted to verify the effectiveness of AHD method with different number of labeled reviews, the results show that AHD can reach very competitive performance with few labeled reviews and large amount of unlabeled reviews. It provides soundness support for the effectiveness of AHD for real applications, where collecting enough unlabeled data is a relatively easy task while it is hard to get enough labeled data.
